# Differences in Diet Quality between School Lunch Participants and Nonparticipants in the United States by Income and Race

**DOI:** 10.3390/nu12123891

**Published:** 2020-12-19

**Authors:** Elizabeth C. Gearan, Kelley Monzella, Leah Jennings, Mary Kay Fox

**Affiliations:** Mathematica Policy Research Inc., 955 Massachusetts Avenue, Suite 801, Cambridge, MA 02139, USA; kmonzella@mathematica-mpr.com (K.M.); ljennings@mathematica-mpr.com (L.J.); mfox@mathematica-mpr.com (M.K.F.)

**Keywords:** National School Lunch Program, dietary intake, nutritional quality, Healthy Eating Index-2010, School Nutrition and Meal Cost Study, race, income

## Abstract

Prior research has shown that participation in the United States’ National School Lunch Program (NSLP) is associated with consuming higher-quality lunches and diets overall, but little is known about differences by income and race/ethnicity. This analysis used 24 h dietary recall data from the School Nutrition and Meal Cost Study to examine how NSLP participation affects the diet quality of students in different income and racial/ethnic subgroups. Diet quality at lunch and over 24 h was assessed using the Healthy Eating Index (HEI)-2010, where higher scores indicate higher-quality intakes. HEI-2010 scores for NSLP participants and nonparticipants in each subgroup were estimated, and two-tailed *t*-tests were conducted to determine whether participant–nonparticipant differences in scores within each subgroup were statistically significant. NSLP participants’ lunches received significantly higher total HEI-2010 scores than those of nonparticipants for lower-income, higher-income, non-Hispanic White, and non-Hispanic Black students, suggesting that participating in the NSLP helps most students consume healthier lunches. These significantly higher total scores for participants’ lunch intakes persisted over 24 h for higher-income students and non-Hispanic White students but not for lower-income students or students of other races/ethnicities. For NSLP participants in all subgroups, the nutritional quality of their 24 h intakes was much lower than at lunch, suggesting that the positive influence of the NSLP on their overall diet quality was negatively influenced by foods consumed the rest of the day (outside of lunch).

## 1. Introduction

On an average school day in the United States, nearly 30 million children, or about half of the student population, participate in the National School Lunch Program (NSLP) [1]. The NSLP is an important safety net for children from low-income households who are eligible to receive meals for free or at a reduced price. Free meals are also provided to children who attend schools in certain low- income areas [2]. The ability to get free or low-cost meals at school eases the financial burden on households that struggle to afford food and ensures that their children have access to healthy food during the school day. NSLP lunches are required to meet nutrition standards that ensure the meals are consistent with the Dietary Guidelines for Americans (DGAs) [3,4].

Prior research has shown that children who participate in the NSLP consume higher-quality diets overall [5,6,7] and also consume healthier lunches than children who bring a lunch from home or get their lunch from other places [5,6,8,9]. Most children’s diets are, however, not consistent with DGA recommendations. Children generally consume more empty calories and sodium [10,11] and fewer fruits, vegetables, and whole grains [10,12] than the DGAs recommend. Poor diet quality in childhood has been linked to nutrient inadequacy—particularly for vitamin D, iron, and potassium [13,14,15,16]—and negative health outcomes, including overweight and obesity [17], prediabetes [18], and mental health conditions [19].

Research has also shown that the nutritional quality of children’s diets varies depending on the child’s race and ethnicity. In particular, several studies have revealed that non-Hispanic Black children had lower-quality diets than Hispanic [20,21,22,23] and non-Hispanic White children [22,23]; and that Hispanic and Mexican-American children had higher quality diets than non-Hispanic White children [21,22]. Other studies have explored the association between children’s diet quality and income, with mixed results. Some studies found that children from higher-income households had higher-quality diets than those from lower-income households [21,24], but other studies reported the opposite [20], or found no consistent pattern of differences between income groups [22,23]. 

Although participating in the NSLP has been associated with higher diet quality, little is known about how it affects the diet quality of children in different income and racial/ethnic subgroups. Using nationally representative data from the School Nutrition and Meal Cost Study (SNMCS), this analysis fills this information gap by exploring whether the positive benefits of participating in the NSLP exist for students of different backgrounds.

## 2. Materials and Methods

### 2.1. Study Design and Data Sources

The SNMCS included nationally representative samples of public school food authorities (SFAs) in the 48 contiguous states and the District of Columbia that participated in the NSLP, non-charter schools within these SFAs (charter schools are independently operated), and students who attended these schools [25]. Within 293 of the schools participating in the SNMCS, data were collected from 2165 students and their parents or guardians (approximately 7 students per school) in the 2014–2015 school year. This analysis is based on 24 h dietary recalls conducted with students aged 6 to 19 years in the spring of 2015. The dietary recalls were administered by trained interviewers using the United States Department of Agriculture’s (USDA’s) Automated Multiple-Pass Method [26] and collected detailed information on all foods and beverages consumed over 24 h on a school day. Data collection across schools was spread across each day of the week (Monday through Friday) to reflect an average school day. Middle- and high-school students completed the dietary recall in one in-person interview, reporting on the previous day’s intake. Elementary school students completed the dietary recall in two separate interviews. The first interview was conducted in person, as soon as possible after students’ lunch periods, and collected information about foods and beverages consumed from awakening through lunch. The second interview was conducted by telephone with parental assistance, usually the following day, and collected information about foods and beverages consumed the rest of the 24 h period.

The 24 h dietary recall data included nutrient values from the Food and Nutrient Database for Dietary Studies, version 2011–2012 [27], and amounts of USDA Food Pattern components (referred to as food groups) from the Food Patterns Equivalents Database, version 2011–2012 [28]. For foods that students obtained from school meals, the study incorporated nutrient and food group values for the foods actually included in NSLP lunches. This process ensured that the nutrient and food group content of these foods were accurately represented in students’ dietary intake data. In addition, using established procedures and information about the names of eating occasions and the time of day at which foods were consumed [29], the study identified foods consumed at lunch. For NSLP participants, lunch intakes could include additional foods that were not part of the school lunch—for example, foods brought from home, obtained outside of school, or purchased at school (such as from a vending machine or snack bar).

This analysis also drew on two other SNMCS data sources. Data on students’ household income and race or ethnicity were obtained from interviews conducted with the parents/guardians, and data on students’ participation in the NSLP on the day covered in the 24 h dietary recall were obtained primarily from school administrative data. When administrative data were not available (for 9 percent of students), the study imputed NSLP participation status using established procedures that consider the types of foods students consumed and the source of those foods [25,29].

The Office of Management and Budget and the New England Institutional Review Board approved the protocol for the SNMCS. The study also followed any institutional review processes a school district required. Passive or active consent (depending on the local school district’s requirements) was obtained from parents and students before collecting the 24-h dietary recall data. The methodology report for the SNMCS describes in detail the study’s design, as well as sampling, recruitment, data collection, and data processing procedures [30]. Additional details about collection and analysis of the data used in this paper are available in Volume 4 of the SNMCS final report [25].

### 2.2. Measuring the Nutritional Quality of Students’ Diets 

This analysis used the Healthy Eating Index (HEI)-2010 to examine the nutritional quality of students’ diets at lunch and over 24 h. The HEI-2010 measures nutritional quality by assessing how well a set of foods aligns with the 2010 DGAs [31]. The HEI is updated to correspond to each new release of the DGA. The HEI-2010 was used for the analysis because the 2010 DGAs were in place when data for the SNMCS were collected [4]. The HEI-2010 has 12 components—9 adequacy components for foods and dietary components that are encouraged and 3 moderation components for dietary components that should be limited or consumed in small amounts (Table 1) [32]. Each component has standards for achieving the maximum possible score (which ranges from 5 to 20) and for receiving the minimum score of 0. The standards are expressed as densities (typically as amounts per 1000 calories). The density-based standards allow quality to be assessed separately from quantity, which means higher scores cannot be achieved simply by consuming larger amounts of food. Densities between the minimum and maximum standards are scored proportionately. Scores for each component are summed to arrive at the total HEI-2010 score, which has a maximum possible score of 100 and is an overall measure of diet quality. Higher scores for each component and for the total score indicate higher nutritional quality and better conformance with the DGAs. For the adequacy components, higher scores reflect higher intakes; for the moderation components, higher scores reflect lower intakes.

### 2.3. Defining Income and Race/Ethnicity Subgroups 

Students were placed in income and race/ethnicity subgroups using data reported by their parent or guardian. Students were categorized as living in households with lower incomes or higher incomes based on their household income relative to the federal poverty level and eligibility for receiving free or reduced-price school meals. Students in the lower-income group had household incomes less than or equal to 185 percent of the federal poverty level and were eligible for free or reduced-price meals; students in the higher-income group had household incomes above 185 percent of the poverty level and were not eligible for free or reduced-price meals. Race/ethnicity subgroups included non-Hispanic White, non-Hispanic Black, Hispanic, and other races (including Asian, American Indian or Alaska Native, Native Hawaiian or other Pacific Islander; and students of multiple or other races).

### 2.4. Statistical Methods 

Mean HEI-2010 scores were estimated using the publicly available code from the National Cancer Institute [33]. Scores were estimated using the population ratio method, which is the preferred method for estimating scores based on a single day of dietary intake data [34]. The HEI-2010 uses Monte Carlo simulation to simulate the densities for 10,000 samples to allow stable estimation of the standard errors. Mean HEI-2010 scores were estimated for students’ lunch intakes (all foods consumed at lunch) and for 24 h intakes (all foods consumed over the 24 h period covered in the dietary recall). Scores were estimated separately for NSLP participants and nonparticipants within each income and race/ethnicity subgroup. Students who had missing information for income or race/ethnicity were excluded from the respective analyses (*n* = 19 for income and *n* = 150 for race/ethnicity). In addition, nonparticipants who did not consume a lunch were excluded from the analysis (*n* = 68). Because the maximum possible scores vary across HEI-2010 components, mean scores are expressed as a percentage of the maximum possible scores. (Supplementary Tables S1–S4 present 95% confidence intervals for all estimates, in addition to mean scores.) Within each income and race/ethnicity subgroup, two-tailed *t*-tests were used to determine whether differences between NSLP participants and nonparticipants were significant at the *p* < 0.05 level.

Table 2 shows demographic characteristics the overall student sample and for NSLP participants and nonparticipants. The table documents the fact that there were statistically significant differences between key demographic characteristics, including gender, age, school level, race/ethnicity, and household income.

To control for these differences in student characteristics that might affect participation in the NSLP, this analysis used inverse probability weighting (IPW). Separate IPW weights were developed for the income and race/ethnicity subgroups using the procedures employed in the SNMCS [25]. The IPW weights ensured that, on average, NSLP participants and nonparticipants in each income and race/ethnicity subgroup were similar in terms of observable student characteristics that could influence participation (for example, household income, age, gender, race/ethnicity, eating habits (such as picky eating and amount student eats relative to others of the same age), and overall health). To implement IPW, covariate-balancing propensity scores were first estimated for each sampled student, which jointly maximized (a) the predicted probability of students’ participation status, and (b) the post-weighted balance of covariates (which included 44 student characteristics) between participants and post-weighted nonparticipants [35]. Estimated propensity scores for NSLP participation were then used to develop IPW weights that balanced NSLP participants and nonparticipants along observable characteristics. 

After IPW weights were applied, the sociodemographic characteristics shown in Table 2 generally exhibited balance (defined as a standardized difference of less than 10 percent between NSLP participants and nonparticipants). For higher-income students, characteristics of NSLP participants and nonparticipants were balanced along all 44 characteristics considered in developing the IPW weights. The same was true for lower-income students except for one of the 44 characteristics. For subgroups defined by race/ethnicity, between 4 characteristics (non-Hispanic White) and 11 characteristics (other races) exhibited imbalance, commonly including gender or income. Due to imbalance on the IPW weights for students in the “other races” subgroup, students in this subgroup were excluded from analyses focused on race/ethnicity (*n* = 181). The final weights used in the analysis were constructed as the product of the IPW weight and student-level sampling weights that accounted for differences between the study sample and the reference population, the study’s complex sample design, and nonresponse [30,36]. This weighting approach adjusts for differences in student characteristics while maintaining fidelity with the population ratio method of calculating HEI scores. 

## 3. Results

### 3.1. Healthy Eating Index-2010 Scores by Household Income 

#### 3.1.1. Lunch Intakes 

**Figure 1:**
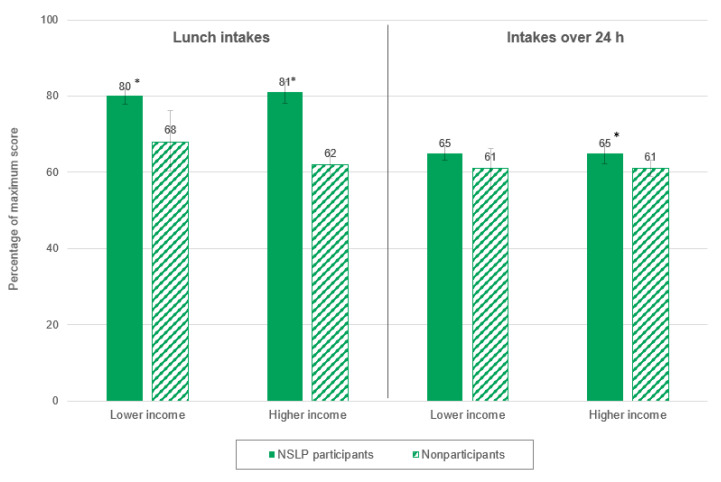
Differences between NSLP participants and nonparticipants in total HEI-2010 scores at lunch and over 24 h, by household income. * Within an income subgroup, the difference between NSLP participants and nonparticipants was significantly different from zero at the *p* < 0.05 level. HEI = Healthy Eating Index; NSLP = National School Lunch Program.

In both the lower-income and higher-income groups, lunches consumed by NSLP participants had significantly higher total HEI-2010 scores than lunches consumed by nonparticipants (for lower-income students, 80 percent of the maximum score for NSLP participants compared with 68 percent for nonparticipants; and for higher-income students, 81 percent for NSLP participants compared with 62 percent for nonparticipants; Figure 1). 

In both income groups, lunches consumed by NSLP participants had significantly higher HEI-2010 scores for total vegetables, whole grains, dairy, refined grains, and empty calories than lunches consumed by nonparticipants (Table 3). In addition, in the lower-income group, lunches consumed by NSLP participants received a significantly lower score for seafood and plant proteins, and in the higher-income group, lunches consumed by NSLP participants received a significantly higher score for sodium. (Higher scores for the moderation components—refined grains, empty calories, and sodium—reflect lower concentrations in students’ intakes). 

#### 3.1.2. Intakes over 24 H 

In the group of students from higher-income households only, NSLP participants had a significantly higher mean total HEI-2010 score over 24 h than nonparticipants did (65 percent versus 61 percent; Figure 1). (The difference for the lower-income group was not statistically significant.) In both income groups, 24 h intakes of NSLP participants had significantly higher scores for whole grains and dairy than those of nonparticipants (Table 4). In the higher-income group, 24 h intakes of NSLP participants also had significantly higher scores for refined grains and sodium than nonparticipants did, but they had significantly lower scores for greens and beans. 

### 3.2. Healthy Eating Index-2010 Scores by Student Race and Ethnicity 

#### 3.2.1. Lunch Intakes 

**Figure 2:**
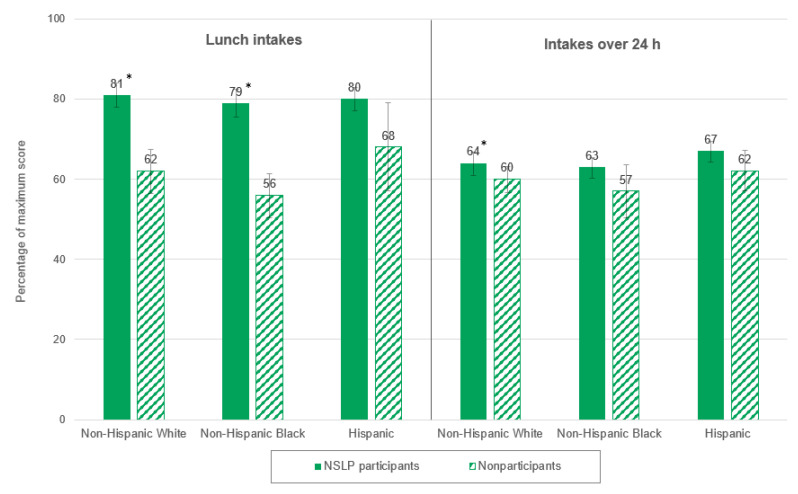
Differences between NSLP participants and nonparticipants in total HEI-2010 scores at lunch and over 24 h, by race/ethnicity. * Within a race/ethnicity subgroup, the difference between NSLP participants and nonparticipants was significantly different from zero at the *p* < 0.05 level. HEI = Healthy Eating Index; NSLP = National School Lunch Program.

For both non-Hispanic White and non-Hispanic Black students, lunches consumed by NSLP participants had significantly higher total HEI-2010 scores than those consumed by nonparticipants (81 percent versus 62 percent of the maximum score, and 79 percent versus 56 percent, respectively; Figure 2). The pattern was similar for Hispanic students, but the difference between NSLP participants and nonparticipants was not statistically significant (80 percent versus 68 percent). 

In all three race/ethnicity subgroups, lunches consumed by NSLP participants had significantly higher scores for dairy, refined grains, and empty calories than lunches consumed by nonparticipants (Table 5). In addition, among non-Hispanic White students and non-Hispanic Black students, NSLP participants had significantly higher scores for whole grains. Finally, among non-Hispanic White students (only), lunches consumed by NSLP participants had a significantly higher score for total vegetables and a significantly lower score for seafood and plant proteins.

#### 3.2.2. Intakes over 24 H

For non-Hispanic White students only, NSLP participants had a significantly higher mean total HEI-2010 score over 24 h than nonparticipants (64 percent versus 60 percent; Figure 2). In all three race/ethnicity subgroups, the 24 h intakes of NSLP participants had significantly higher scores for dairy than those of nonparticipants (Table 6). In addition, among non-Hispanic White students, 24 h intakes of NSLP participants had significantly higher scores for total vegetables, whole grains, and refined grains than nonparticipants. Among non-Hispanic Black students, 24 h intakes of NSLP participants had a significantly lower score for greens and beans than nonparticipants. Finally, among Hispanic students, NSLP participants had a significantly higher score for whole grains than nonparticipants. 

## 4. Discussion

The results of this nationally representative analysis of students’ diets reveal that lunches consumed by NSLP participants are of higher nutritional quality than lunches consumed by nonparticipants for students from both lower- and higher-income households and for both non-Hispanic White and non-Hispanic Black students. A similar trend was observed for Hispanic students, but the difference in total HEI-2010 scores between participants and nonparticipants was not statistically significant for this subgroup. This might be driven by the fact that the lunches consumed by Hispanic nonparticipants were healthier than lunches consumed by nonparticipants in the other race/ethnicity subgroups. As shown in Figure 2, lunches consumed by Hispanic nonparticipants received a higher total HEI-2010 score than lunches consumed by non-Hispanic White and non-Hispanic Black nonparticipants (differences were not tested for statistical significance). This pattern is consistent with prior studies that found that Hispanic children’s diets, in general, receive higher total HEI scores than those of children in other race/ethnicity groups [20,22]. With the exception of Hispanic students, findings from this analysis suggest that participating in the NSLP helps students consume lunches that are more aligned with DGA recommendations than lunches brought from home or obtained elsewhere. This is consistent with findings from previous research that has shown a positive relationship between student participation in the NSLP and consumption of healthier lunches [5,6,8,9].

Total HEI-2010 scores for NSLP participants in all subgroups dropped considerably from lunch to 24 h (by 13 to 17 percentage points across all subgroups of NSLP participants; see Figure 1 and Figure 2), which suggests that the foods these students consumed the rest of the day (outside of lunch) had a negative influence on their overall diets. (The same trend was observed for nonparticipants in most subgroups, but the drop in scores was smaller in magnitude—between 1 and 7 percentage points). Other analyses of SNMCS data examined the nutritional quality of NSLP participants’ intakes from school meals versus intakes outside of school meals and found that the nutritional quality of foods consumed outside of school meals was substantially lower [37]. Over 24 h, the positive, significant differences between NSLP participants and nonparticipants in total HEI-2010 scores remained significant for only two subgroups: higher-income students and non-Hispanic White students. For NSLP participants in these two subgroups, most of the positive significant differences observed for HEI components at lunch persisted over 24 h (5 of the 6 components for higher-income NSLP participants, and 4 of the 5 components for non-Hispanic White students). However, this was true for fewer components for lower-income NSLP participants and non-Hispanic Black NSLP participants (where the positive differences in scores for 2 of the 5 components and 1 of the 4 components, respectively, remained significant over 24 h). This may suggest that foods consumed by these NSLP participants during the rest of the day had a larger negative influence on their overall diets compared to non-Hispanic White and higher-income NSLP participants. Another potential explanation for the differences observed at lunch not persisting over 24 h for some subgroups of students is that the contribution of a single meal (in this case, lunch) may not be large enough to influence the overall nutritional quality of diets over 24 h. It has been estimated that NSLP participants consume less than 30% of their daily calories at lunch [25,38]. Finally, as discussed previously, Hispanic children tend to have higher-quality diets than children in other race/ethnicity subgroups, so the lack of significant differences between Hispanic NSLP participants and nonparticipants over 24 h is not surprising [20,22]. 

Differences in total HEI-2010 scores were driven by differences in HEI-2010 component scores. Across all subgroups defined by income or race/ethnicity, NSLP participants’ lunch intakes had significantly higher scores for dairy, refined grains, and empty calories; and, with the exception of Hispanic students, they also had higher scores for whole grains. In addition, NSLP participants in both income groups and non-Hispanic White participants had significantly higher scores for total vegetables. While there was some variation across subgroups, the positive, significant differences observed for these components indicate that NSLP participants’ lunches had higher concentrations of dairy, whole grains, and vegetables, and had lower concentrations of refined grains and empty calories. These are important differences given the role these components can play in school-age children’s diets and health. For example, higher intakes of whole grains have been associated with lower body weight and may play a role in reducing the risk of heart disease, cancer, and Type 2 diabetes [39,40,41] while adequate dairy consumption is associated with greater intake of vitamin D, calcium, and protein, which could improve bone mineral content [42]. Additionally, limiting children’s consumption of empty calories (which are calories from solid fats and added sugars) is particularly important because excessive intakes of empty calories can displace more nutrient-dense foods and increase calorie intakes beyond required levels [4,43]. 

Differences in HEI component scores for lunches consumed by NSLP participants and nonparticipants might reflect policy changes that were in effect when data for the SNMCS were collected. In school year 2014–2015, school lunches were required to meet updated nutrition standards that were designed to ensure the meals aligned with DGA recommendations [3]. For example, the updated standards required the inclusion of whole-grain-rich foods, larger portion sizes and greater variety of vegetables, and limited fluid milk to nonfat varieties and plain low-fat (1%) milk. Findings for specific HEI components suggest that the lunches consumed by NSLP participants reflected these updated requirements and that the NSLP provided students with access to high-quality foods that they may not otherwise consume at lunch, while at the same time limiting their access to lower-quality foods (for example, those with refined grains and/or empty calories). Given the positive influence of the NSLP on the nutritional quality of students’ lunch intakes, policymakers, program administrators, and schools should explore ways to increase participation in the program. When data for the SNMCS were collected, only 56% of students participated in the NSLP on an average day [25]. Given that student participation in the NSLP has also been associated with a reduction in food insecurity, efforts to increase participation should focus on expanding and encouraging participation among children who are more likely to live in food-insecure households, including non-Hispanic Blacks, Hispanics, and children from lower-income families [44,45].

Changes to the foods school-age children consume outside of lunch can further improve the nutritional quality of their overall diets. Intakes of vegetables and sodium are of particular concern. For all subgroups of students, HEI scores were generally at or below 50% of the maximum possible score for total vegetables, greens, and beans, and sodium. It is important to improve these scores because adequate vegetable intake is associated with lower risk for chronic conditions such as obesity, hypertension, coronary heart disease, and cancer [46], and excessive sodium intake is associated with elevated risk of high blood pressure during childhood and into adulthood [47,48]. To improve overall diet quality and alignment with DGA recommendations, nutrition education efforts and interventions with school-age children and their parents should target these dietary components and emphasize the importance of consuming healthy foods throughout the day. 

This analysis has several notable strengths. The SNMCS data used in the analysis included a nationally representative sample of students, identified NSLP participants based on administrative data, and used information on the actual nutrient content of foods obtained from reimbursable meals to ensure these foods were accurately represented in students’ dietary intake data [25,30]. In addition, the analysis used a weighting approach (IPW) to minimize the differences between NSLP participants and nonparticipants on characteristics that could influence participation, making it less likely that differences in HEI-2010 scores reflect underlying differences between participants and nonparticipants [35]. Finally, it explored an important information gap in how school meal participation affects the diet quality of students based on their household’s income or their race/ethnicity.

Nonetheless, the analysis does have limitations. First, it made many comparisons between NSLP participants and nonparticipants, which may have resulted in inflated Type 1 error rates. Multiple-comparison adjustments were not made due to the exploratory nature of the analysis. Few studies have previously explored the association between school lunch participation and HEI scores based on students’ household income or their race/ethnicity. In addition, there were relatively small sample sizes for some subgroups of students—particularly non-Hispanic Black students and Hispanic students—which can limit the ability to detect significant differences in HEI-2010 scores within subgroups. Additional studies with larger samples of non-Hispanic Black students and Hispanic students—as well as analyses including students in other or mixed-race categories, which were excluded here—are needed to fully investigate whether there are differences in the relationship between NSLP participation and diet quality.

## 5. Conclusions 

The NSLP is designed to provide school-age children with access to nutritious meals on school days. Findings from this analysis show that lunches consumed by NSLP participants in most income and race/ethnicity subgroups were of higher nutritional quality than those consumed by nonparticipants. Policymakers, program administrators, and schools should explore ways to increase participation in the NSLP so that more children benefit from the positive influence of the program on lunch intakes. For NSLP participants in all income and race/ethnicity subgroups, the nutritional quality of their 24 h intakes was much lower than that observed at lunch, suggesting that the positive influence of the NSLP on overall diet quality was negatively influenced by foods students consumed the rest of the day (outside of lunch). Nutrition education efforts and interventions with school-age children and their parents should emphasize the importance of consuming healthy foods throughout the day to improve overall diet quality. 

## Figures and Tables

**Table 1:** Healthy Eating Index (HEI)-2010 components and standards for scoring.

	Maximum Score	Standard for Maximum Score ^a^	Standard for Minimum Score of Zero ^a^
Adequacy components: Higher scores reflect higher concentrations in students’ diets			
Total fruit ^b^	5	≥0.8 c equivalent per 1000 kcal	No fruit
Whole fruit ^c^	5	≥0.4 c equivalent per 1000 kcal	No whole fruit
Total vegetables ^d^	5	≥1.1 c equivalent per 1000 kcal	No vegetables
Greens and beans ^d^	5	≥0.2 c equivalent per 1000 kcal	No dark green vegetables, beans, or peas
Whole grains	10	≥1.5 oz equivalent per 1000 kcal	No whole grains
Dairy ^e^	10	≥1.3 c equivalent per 1000 kcal	No dairy
Total protein foods ^f^	5	≥2.5 oz equivalent per 1000 kcal	No protein foods
Seafood and plant proteins ^f,g^	5	≥0.8 oz equivalent per 1000 kcal	No seafood or plant proteins
Fatty acids ^h^	10	(PUFAs ^i^ + MUFAs ^j^)/saturated fatty acids ≥ 2.5	(PUFAs + MUFAs)/saturated fatty acids ≤ 1.2
Moderation components: Higher scores reflect lower concentrations in students’ diets			
Refined grains	10	≤1.8 oz equivalent per 1000 kcal	≥4.3 oz equivalent per 1000 kcal
Sodium	10	≤1.1 g per 1000 kcal	≥2.0 g per 1000 kcal
Empty calories ^k^	20	≤19% of energy	≥50% of energy
Total Score	100		

Adapted from USDA (United States Department of Agriculture) 2013 [32]. ^a^ Concentrations between the minimum and maximum standard are scored proportionately. Higher scores reflect higher nutritional quality. ^b^ Includes 100 percent fruit juice. ^c^ Includes all forms except juice. ^d^ Includes any beans and peas not counted as total protein foods. ^e^ Includes all milk products, such as fluid milk, yogurt, cheese, and fortified soy beverages. ^f^ Beans and peas are included here (and not with vegetables) when the total protein foods standard is otherwise not met. ^g^ Includes seafood, nuts, seeds, soy products (other than beverages), and beans and peas counted toward total protein foods. ^h^ Ratio of polyunsaturated and monounsaturated fatty acids (PUFAs and MUFAs) to saturated fatty acids. ^i^ PUFA = polyunsaturated fatty acid. ^j^ MUFA = monounsaturated fatty acid. ^k^ Kcals from solid fats, added sugars, and alcoholic beverages. Threshold for counting alcohol is >13 g/1000 kcal. c: cup; g: gram; kcal: calorie; oz: ounce.

**Table 2:** Sociodemographic characteristics of students, by National School Lunch Program (NSLP) participation status.

Characteristics	All Students (*n* = 2097)	NSLP Participants (*n* = 1254)	Nonparticipants (*n* = 843)
Gender (%)			
Male	50.4	53.2	46.7 *
Age (mean years)	12.1	11.2	13.1 *
School level (%)			
Elementary	45.4	56.8	30.0 *
Middle	20.7	19.3	22.6
High	33.9	23.9	47.3 *
Race/ethnicity (%)			
White, non-Hispanic	48.9	42	58.1 *
Hispanic	24.4	30.3	16.4 *
Black, non-Hispanic	12.4	15.7	8.0 *
Multiracial/other	8	7.1	9.2
Missing	6.3	4.8	8.2 *
Household income as percentage of federal poverty level (%)			
Eligible for free or reduced-price meals (≤185%)	42.5	59.7	19.5 *
Not eligible for free or reduced-price meals (>185%)	55.8	38.9	78.3 *
Missing	1.8	1.6	2.1

* Difference between NSLP participants and nonparticipants was significantly different from zero at *p* < 0.05 level. NSLP: National School Lunch Program.

**Table 3:** Differences between NSLP participants and nonparticipants in mean HEI-2010 scores for lunch intakes, by household income.

	Lower-Income Students			Higher-Income Students		
	NSLP Participants (*n* = 757)	Nonparticipants (*n* = 210)	Difference (NSLP Participants − Nonparticipants)	NSLP Participants (*n* = 475)	Nonparticipants (*n* = 614)	Difference (NSLP Participants − Nonparticipants)
Adequacy components: Higher scores reflect higher concentrations in students’ lunch intakes						
Total fruit	100.0	82.6	17.4	98.5	99.6	−1.1
Whole fruit	100.0	98.4	1.6	100.0	100.0	0.0
Total vegetables	55.1	33.5	21.6 *	48.9	35.8	13.1 *
Greens and beans	35.3	26.9	8.5	12.1	26.5	−14.4
Whole grains	100.0	61.6	38.4 *	100.0	61.0	39.0 *
Dairy	100.0	69.5	30.5 *	100.0	64.5	35.5 *
Total protein foods	99.0	99.5	−0.5	98.1	99.9	−1.9
Seafood and plant proteins	39.6	94.9	−55.3 *	77.4	86.7	−9.3
Fatty acids	60.3	72.0	−11.7	69.2	58.4	10.8
Moderation components: Higher scores reflect lower concentrations in students’ lunch intakes						
Refined grains	91.3	48.8	42.5 *	82.7	26.9	55.7 *
Sodium	40.2	54.5	−14.3	45.7	27.7	17.9 *
Empty calories	95.7	77.9	17.8 *	94.9	79.2	15.7 *
Total HEI score	79.8	68.0	11.8 *	80.5	62.1	18.4 *

Notes: Mean HEI-2010 scores are expressed as a percentage of maximum possible scores. * Within an income subgroup, the difference between NSLP participants and nonparticipants was significantly different from zero at the *p* < 0.05 level. HEI = Healthy Eating Index; NSLP = National School Lunch Program.

**Table 4:** Differences between NSLP participants and nonparticipants in mean HEI-2010 scores for 24 h intakes, by household income.

	Lower-Income Students			Higher-Income Students		
	NSLP Participants (*n* = 757)	Nonparticipants (*n* = 210)	Difference (NSLP Participants − Nonparticipants)	NSLP Participants (*n* = 475)	Nonparticipants (*n* = 614)	Difference (NSLP Participants − Nonparticipants)
Adequacy components: Higher scores reflect higher concentrations in students’ 24 h intakes						
Total fruit	95.9	77.2	18.7	80.4	93.8	−13.4
Whole fruit	100	93.7	6.3	99.5	100	−0.5
Total vegetables	46.2	46.2	0	42.2	41.4	0.8
Greens and beans	17.1	30.7	−13.6	13.1	33.1	−20.0 *
Whole grains	57.4	41.4	16.0 *	58	43.4	14.5 *
Dairy	98.5	79.6	18.9 *	97.7	82	15.7 *
Total protein foods	96.1	99.1	−3.0	97.7	98.7	−1.1
Seafood and plant proteins	59.6	81.5	−21.9	71	73.1	−2.1
Fatty acids	41.2	43.3	−2.0	37.3	38.6	−1.2
Moderation components: Higher scores reflect lower concentrations in students’ 24 h intakes						
Refined grains	57.9	53.1	4.7	62.1	41.9	20.2 *
Sodium	45.4	50.7	−5.3	48.8	38.8	10.0 *
Empty calories	72.5	65.9	6.6	72	72.3	−0.3
Total HEI score	65.3	61.4	3.9	65	60.9	4.0 *

Notes: Mean HEI-2010 scores are expressed as a percentage of maximum possible scores. * Within an income subgroup, the difference between NSLP participants and nonparticipants was significantly different from zero at the *p* < 0.05 level. HEI = Healthy Eating Index; NSLP = National School Lunch Program.

**Table 5:** Differences between NSLP participants and nonparticipants in mean HEI-2010 scores for lunch intakes, by race/ethnicity.

	Non-Hispanic White Students			Non-Hispanic Black Students			Hispanic Students		
	NSLP Participants (*n* = 485)	Nonparticipants (*n* = 442)	Difference (NSLP Participants − Nonparticipants)	NSLP Participants (*n* = 183)	Nonparticipants (*n* = 74)	Difference (NSLP Participants − Nonparticipants)	NSLP Participants (*n* = 376)	Nonparticipants (*n* = 161)	Difference (NSLP Participants −Nonparticipants)
Adequacy components: Higher scores reflect higher concentrations in students’ lunch intakes									
Total fruit	99.5	92.2	7.4	99.7	92.6	7.1	100.0	96.3	3.7
Whole fruit	100.0	100.0	0.0	100.0	99.9	0.1	100.0	99.9	0.1
Total vegetables	56.3	30.1	26.3 *	34.9	27.2	7.7	51.4	50.8	0.6
Greens and beans	29.0	16.3	12.7	19.1	19.5	−0.4	12.9	37.3	−24.4
Whole grains	99.9	53.5	46.4 *	99.2	68.2	31.0 *	100.0	74.4	25.6
Dairy	100.0	65.0	35.0 *	99.9	60.6	39.2 *	100.0	75.1	24.9 *
Total protein foods	97.2	99.3	−2.1	99.2	99.7	−0.4	91.2	96.9	−5.7
Seafood and plant proteins	67.3	99.6	−32.3 *	50.6	34.7	15.8	52.2	63.7	−11.4
Fatty acids	62.1	67.1	−5.0	68.2	46.6	21.6	67.7	55.0	12.7
Moderation components: Higher scores reflect lower concentrations in students’ lunch intakes									
Refined grains	86.8	22.1	64.7 *	94.8	37.8	57.0 *	88.0	60.4	27.5 *
Sodium	45.4	38.2	7.1	35.3	14.5	20.8	42.7	43.5	−0.8
Empty calories	93.2	76.5	16.7 *	95.0	72.1	22.8 *	98.5	76.5	22.0 *
Total HEI score	80.5	61.8	18.8 *	78.9	55.9	23.0 *	79.9	68.4	11.5

Notes: Mean HEI-2010 scores are expressed as a percentage of maximum possible scores. * Within a race/ethnicity subgroup, the difference between NSLP participants and nonparticipants was significantly different from zero at the *p* < 0.05 level. HEI = Healthy Eating Index; NSLP = National School Lunch Program.

**Table 6:** Differences between NSLP participants and nonparticipants in mean HEI-2010 scores for 24 h intakes, by race/ethnicity.

	Non-Hispanic White Students			Non-Hispanic Black Students			Hispanic Students		
	NSLP Participants (*n* = 485)	Nonparticipants (*n* = 442)	Difference (NSLP Participants − Nonparticipants)	NSLP Participants (*n* = 183)	Nonparticipants (*n* = 74)	Difference (NSLP Participants − Nonparticipants)	NSLP Participants (*n* = 376)	Nonparticipants (*n* = 161)	Difference (NSLP Participants − Nonparticipants)
Adequacy components: Higher scores reflect higher concentrations in students’ 24 h intakes									
Total fruit	78.9	76.8	2.1	97.1	81.0	16.1	98.7	92.3	6.4
Whole fruit	99.3	99.2	0.1	95.5	86.6	8.9	100.0	99.7	0.3
Total vegetables	45.9	36.1	9.7 *	40.7	47.7	−7.0	44.9	55.7	−10.8
Greens and beans	13.9	22.8	−8.9	18.3	73.6	−55.2 *	11.5	23.2	−11.7
Whole grains	54.7	38.5	16.2 *	49.1	51.6	−2.5	68.5	39.1	29.4 *
Dairy	99.3	84.7	14.5 *	79.5	58.0	21.6 *	100.0	84.2	15.7 *
Total protein foods	94.7	97.9	−3.2	99.8	93.5	6.2	93.3	94.5	−1.3
Seafood and plant proteins	67.2	82.8	−15.6	49.5	27.6	21.9	69.3	84.9	−15.6
Fatty acids	35.9	40.6	−4.7	51.6	44.8	6.8	40.3	35.4	4.9
Moderation components: Higher scores reflect lower concentrations in students’ 24 h intakes									
Refined grains	63.0	42.4	20.6 *	65.9	47.3	18.7	53.7	46.9	6.9
Sodium	50.8	48.0	2.8	46.0	38.7	7.3	42.7	44.9	−2.2
Empty calories	68.2	66.8	1.3	70.4	62.8	7.6	79.9	72.9	7.0
Total HEI score	64.0	59.6	4.4 *	63.3	57.1	6.3	67.4	62.1	5.2

Notes: Mean HEI-2010 scores are expressed as a percentage of maximum possible scores. * Within a race/ethnicity subgroup, the difference between NSLP participants and nonparticipants was significantly different from zero at the *p* < 0.05 level. HEI = Healthy Eating Index; NSLP = National School Lunch Program.

## Supplementary Materials

The following are available online at https://www.mdpi.com/2072-6643/12/12/3891/s1, Table S1. Differences between NSLP participants and nonparticipants in mean HEI-2010 scores for lunch intakes, by household income (including 95% CIs); Table S2. Differences between NSLP participants and nonparticipants in mean HEI-2010 scores for 24 h intakes, by household income (including 95% CIs); Table S3. Differences between NSLP participants and nonparticipants in mean HEI-2010 scores for lunch intakes, by race/ethnicity (including 95% CIs); Table S4. Differences between NSLP participants and nonparticipants in mean HEI-2010 scores for 24 h intakes, by race/ethnicity (including 95% CIs).
