# Supplementary material for: Differences in Diet Quality between School Lunch Participants and Nonparticipants in the United States by Income and Race
Source: Nutrients. 2020 Dec 19;12(12):3891. doi: 10.3390/nu12123891 (PMC7765856; doi:10.3390/nu12123891)
Supplement: Supplementary file 1 [file nutrients-12-03891-s001.pdf]

nutrients

MDPI

Table 1. Differences between NSLP participants and nonparticipants in mean HEI-2010 scores for lunch intakes, by household income (including 95% CIs).

	Lower-Income Students			Higher-Income Students		
	**NSLP Participants (*n* = 757)**	**Nonparticipants (*n* = 210)**	**Difference (NSLP Participants – Nonparticipants)**	**NSLP Participants (*n* = 475)**	**Nonparticipants (*n* = 614)**	**Difference (NSLP Participants – Nonparticipants)**
Adequacy components: Higher scores reflect higher concentrations in students' lunch intakes						
Total fruit	100.0 (100.0, 100.0)	82.6 (54.8, 100.0)	17.4	98.5 (88.5, 100.0)	99.6 (93.8, 100.0)	-1.1
Whole fruit	100.0 (100.0, 100.0)	98.4 (78.1, 100.0)	1.6	100.0 (100.0, 100.0)	100.0 (100.0, 100.0)	0.0
Total vegetables	55.1 (47.7, 62.2)	33.5 (21.7, 47.3)	21.6*	48.9 (40.6, 57.2)	35.8 (30.6, 40.9)	13.1*
Greens and beans	35.3 (18.1, 51.1)	26.9 (11.5, 42.9)	8.5	12.1 (4.0, 23.6)	26.5 (10.0, 42.5)	-14.4
Whole grains	100.0 (100.0, 100.0)	61.6 (39.3, 89.2)	38.4*	100.0 (100.0, 100.0)	61.0 (49.6, 72.5)	39.0*
Dairy	100.0 (100.0, 100.0)	69.5 (54.6, 86.8)	30.5*	100.0 (100.0, 100.0)	64.5 (56.4, 72.8)	35.5*
Total protein foods	99.0 (88.5, 100.0)	99.5 (91.9, 100.0)	-0.5	98.1 (90.7, 100.0)	99.9 (99.9, 100.0)	-1.9
Seafood and plant proteins	39.6 (23.7, 56.8)	94.9 (40.2, 100.0)	-55.3*	77.4 (55.8, 98.5)	86.7 (65.6, 100.0)	-9.3
Fatty acids	60.3 (50.5, 70.4)	72.0 (50.2, 92.3)	-11.7	69.2 (55.3, 84.3)	58.4 (48.8, 68.7)	10.8
Moderation components: Higher scores reflect lower concentrations in students' lunch intakes						
Refined grains	91.3 (81.8, 100.0)	48.8 (21.6, 82.3)	42.5*	82.7 (73.5, 91.8)	26.9 (14.4, 39.7)	55.7*
Sodium	40.2 (31.3, 49.1)	54.5 (39.1, 68.7)	-14.3	45.7 (36.7, 54.3)	27.7* (16.2, 38.8)	17.9*
Empty calories	95.7 (91.2, 100.0)	77.9 (66.7, 88.5)	17.8*	94.9 (89.6, 100.0)	79.2 (74.2, 84.4)	15.7*
Total HEI score	79.8 (77.7, 81.7)	68.0 (60.3, 76.1)	11.8*	80.5 (77.5, 83.4)	62.1 (58.6, 65.7)	18.4*

Notes: Mean HEI-2010 scores are expressed as a percentage of maximum possible scores. Upper and lower limits of 95% CIs are included in parentheses below the mean HEI score. * Within an income subgroup, the difference between NSLP participants and nonparticipants was significantly different from zero at the $p < 0.05$ level. CI = confidence interval; HEI = Healthy Eating Index; NSLP = National School Lunch Program.

Table 2. Differences between NSLP participants and nonparticipants in mean HEI-2010 scores for 24 h intakes, by household income (including 95% CIs).

	Lower-Income Students			Higher-Income Students		
	**NSLP Participants (*n* = 757)**	**Nonparticipants (*n* = 210)**	**Difference (NSLP Participants – Nonparticipants)**	**NSLP Participants (*n* = 475)**	**Nonparticipants (*n* = 614)**	**Difference (NSLP Participants – Nonparticipants)**
Adequacy components: Higher scores reflect higher concentrations in students' 24 h						
Total fruit	95.9 (86.9, 100.0)	77.2 (58.8, 97.7)	18.7	80.4 (71.0, 90.2)	93.8 (83.4, 100.0)	-13.4
Whole fruit	100.0 (100.0, 100.0)	93.7 (69.3, 100.0)	6.3	99.5 (93.7, 100.0)	100.0 (100.0, 100.0)	-0.5
Total vegetables	46.2 (41.8, 50.6)	46.2 (37.6, 55.4)	0.0	42.2 (36.0, 48.4)	41.4 (36.5, 46.7)	0.8
Greens and beans	17.1 (11.5, 29.6)	30.7 (10.8, 52.5)	-13.6	13.1 (6.5, 26.4)	33.1* (20.1, 49.1)	-20.0
Whole grains	57.4 (51.5, 63.5)	41.4 (31.0, 52.8)	16.0*	58.0 (51.3, 64.5)	43.4 (38.3, 48.5)	14.5*
Dairy	98.5 (94.1, 100.0)	79.6 (69.5, 90.4)	18.9*	97.7 (91.8, 100.0)	82.0 (76.0, 87.9)	15.7*
Total protein foods	96.1	99.1	-3.0	97.7	98.7	-1.1

	(88.5, 100.0)	(92.2, 100.0)		(90.3, 100.0)	(93.3, 100.0)	
Seafood and plant proteins	59.6<br>(47.2, 70.2)	81.5<br>(46.6, 100.0)	-21.9	71.0<br>(55.7, 86.1)	73.1<br>(59.5, 86.6)	-2.1
Fatty acids	41.2<br>(37.0, 45.6)	43.3<br>(31.4, 55.4)	-2.0	37.3<br>(30.7, 44.4)	38.6<br>(33.2, 44.2)	-1.2
Moderation components: Higher scores reflect lower concentrations in students' 24 h intakes						
Refined grains	57.9<br>(54.3, 61.5)	53.1<br>(41.5, 65.2)	4.7	62.1<br>(54.7, 69.5)	41.9<br>(35.1, 48.7)	20.2*
Sodium	45.4<br>(41.4, 49.4)	50.7<br>(44.3, 56.9)	-5.3	48.8<br>(43.8, 53.7)	38.8*<br>(31.8, 45.8)	10.0*
Empty calories	72.5<br>(68.8, 76.4)	65.9<br>(60.5, 71.6)	6.6	72.0<br>(67.0, 76.8)	72.3<br>(68.0, 76.6)	-0.3
Total HEI score	65.3<br>(63.4, 67.2)	61.4<br>(56.1, 66.6)	3.9	65.0<br>(62.3, 67.5)	60.9<br>(58.9, 63.0)	4.0*

Notes: Mean HEI-2010 scores are expressed as a percentage of maximum possible scores. Upper and lower limits of 95% CIs are included in parentheses below the mean HEI score. * Within an income subgroup, the difference between NSLP participants and nonparticipants was significantly different from zero at the $p < 0.05$ level. CI = confidence interval; HEI = Healthy Eating Index; NSLP = National School Lunch Program.

Table 3. Differences between NSLP participants and nonparticipants in mean HEI-2010 scores for lunch intakes, by race/ethnicity (including 95% CIs).

	**Non-Hispanic White Students**			**Non-Hispanic Black Students**			**Hispanic Students**		
	NSLP Participants (*n* = 485)	Nonparticipants (*n* = 442)	**Difference (NSLP Participants – Nonparticipants)**	**NSLP Participants (*n* = 183)**	**Nonparticipants (*n* = 74)**	**Difference (NSLP Participants – Nonparticipants)**	**NSLP Participants (*n* = 376)**	**Nonparticipants (*n* = 161)**	**Difference (NSLP Participants – Nonparticipants)**
Adequacy components: Higher scores reflect higher concentrations in students' lunch intakes									
Total fruit	99.5 (93.9, 100.0)	92.2 (75.2, 100.0)	7.4	99.7 (96.9, 100.0)	92.6 (66.4, 100.0)	7.1	100.0 (100.0, 100.0)	96.3 (70.7, 100.0)	3.7
Whole fruit	100.0 (100.0, 100.0)	100.0 (100.0, 100.0)	0.0	100.0 (100.0, 100.0)	99.9 (100.0, 100.0)	0.1	100.0 (100.0, 100.0)	99.9 (100.0, 100.0)	0.1
Total vegetables	56.3 (47.4, 65.7)	30.1 (23.5, 37.0)	26.3*	34.9 (24.8, 44.7)	27.2 (13.6, 41.4)	7.7	51.4 (39.5, 63.2)	50.8 (33.8, 67.3)	0.6
Greens and beans	29.0 (13.1, 44.0)	16.3 (2.1, 33.8)	12.7	19.1 (1.3, 38.7)	19.5 (-0.4, 39.2)	-0.4	12.9 (6.4, 20.7)	37.3 (11.3, 65.3)	-24.4
Whole grains	99.9 (100.0, 100.0)	53.5 (39.4, 68.6)	46.4*	99.2 (89.4, 100.0)	68.2 (42.5, 89.5)	31.0*	100.0 (100.0, 100.0)	74.4 (43.4, 100.0)	25.6
Dairy	100.0 (100.0, 100.0)	65.0 (53.9, 76.9)	35.0*	99.9 (98.5, 100.0)	60.6 (46.8, 75.4)	39.2*	100.0 (100.0, 100.0)	75.1 (56.6, 94.8)	24.9*
Total protein foods	97.2 (86.4, 100.0)	99.3 (92.3, 100.0)	-2.1	99.2 (87.5, 100.0)	99.7 (95.0, 100.0)	-0.4	91.2 (79.9, 100.0)	96.9 (81.8, 100.0)	-5.7
Seafood and plant proteins	67.3 (46.2, 88.1)	99.6 (93.8, 100.0)	-32.3*	50.6 (27.1, 73.6)	34.7 (12.0, 60.1)	15.8	52.2 (34.2, 69.8)	63.7 (6.3, 100.0)	-11.4
Fatty acids	62.1 (50.1, 75.2)	67.1 (53.2, 82.6)	-5.0	68.2 (49.1, 87.7)	46.6 (29.8, 65.4)	21.6	67.7 (53.2, 82.7)	55.0 (26.0, 94.3)	12.7
Moderation components: Higher scores reflect lower concentrations in students' lunch intakes									
Refined grains	86.8 (75.0, 98.7)	22.1 (3.7, 41.6)	64.7*	94.8 (80.4, 100.0)	37.8 (10.7, 68.0)	57.0*	88.0 (75.7, 100.0)	60.4 (38.6, 80.7)	27.5*
Sodium	45.4 (35.6, 54.8)	38.2 (21.5, 54.1)	7.1	35.3 (13.2, 55.0)	14.5 (0.0, 37.5)	20.8	42.7 (36.0, 49.8)	43.5 (30.0, 57.6)	-0.8
Empty calories	93.2 (87.9, 98.8)	76.5 (68.1, 85.2)	16.7*	95.0 (87.2, 100.0)	72.1 (59.4, 86.7)	22.8*	98.5 (93.8, 100.0)	76.5 (62.0, 93.8)	22.0*
Total HEI score	80.5 (77.4, 83.5)	61.8 (56.3, 67.3)	18.8*	78.9 (75.4, 82.4)	55.9 (50.2, 61.3)	23.0*	79.9 (77.0, 82.7)	68.4 (57.5, 79.5)	11.5

Notes: Mean HEI-2010 scores are expressed as a percentage of maximum possible scores. Upper and lower limits of 95% CIs are included in parentheses below the mean HEI score. * Within a race/ethnicity subgroup, the difference between NSLP participants and nonparticipants was significantly different from zero at the $p < 0.05$ level. CI = confidence interval; HEI = Healthy Eating Index; NSLP = National School Lunch Program. .

Table 4. Differences between NSLP participants and nonparticipants in mean HEI-2010 scores for 24 h intakes, by race/ethnicity (including 95% CIs).

	Non-Hispanic White Students			Non-Hispanic Black Students			Hispanic Students		
	**NSLP Participants (*n* = 485)**	**Nonparticipants (*n* = 442)**	**Difference (NSLP Participants – Nonparticipants)**	**NSLP Participants (*n* = 183)**	**Nonparticipants (*n* = 74)**	**Difference (NSLP Participants – Nonparticipants)**	**NSLP Participants (*n* = 376)**	**Nonparticipants (*n* = 161)**	**Difference (NSLP Participants – Nonparticipants)**
Adequacy components: Higher scores reflect higher concentrations in students' 24 h intakes									
Total fruit	78.9 (68.7, 89.6)	76.8 (63.7, 90.6)	2.1	97.1 (84.2, 100.0)	81.0 (61.7, 100.0)	16.1	98.7 (91.5, 100.0)	92.3 (69.9, 100.0)	6.4
Whole fruit	99.3 (91.6, 100.0)	99.2 (90.0, 100.0)	0.1	95.5 (76.8, 100.0)	86.6 (51.6, 100.0)	8.9	100.0 (100.0, 100.0)	99.7 (95.9, 100.0)	0.3
Total vegetables	45.9 (39.2, 52.5)	36.1 (31.2, 41.3)	9.7*	40.7 (34.6, 46.4)	47.7 (38.0, 57.3)	-7.0	44.9 (38.6, 51.3)	55.7 (46.9, 64.7)	-10.8
Greens and beans	13.9 (8.1, 24.8)	22.8 (11.5, 36.3)	-8.9	18.3 (10.7, 26.0)	73.6* (26.0, 100.0)	-55.2	11.5 (6.9, 17.4)	23.2 (10.8, 42.1)	-11.7
Whole grains	54.7 (48.6, 60.4)	38.5 (32.1, 45.2)	16.2*	49.1 (39.3, 59.1)	51.6 (22.2, 83.1)	-2.5	68.5 (61.3, 75.7)	39.1 (28.2, 49.8)	29.4
Dairy	99.3 (94.8, 100.0)	84.7 (77.1, 92.7)	14.5*	79.5 (72.8, 86.3)	58.0 (47.6, 67.7)	21.6*	100.0 (99.9, 100.0)	84.2 (72.6, 96.1)	15.7*
Total protein foods	94.7 (85.3, 100.0)	97.9 (89.7, 100.0)	-3.2	99.8 (96.7, 100.0)	93.5 (78.2, 100.0)	6.2	93.3 (84.9, 100.0)	94.5 (82.7, 100.0)	-1.3
Seafood and plant proteins	67.2 (51.8, 81.5)	82.8 (64.9, 100.0)	-15.6	49.5 (32.3, 69.2)	27.6 (14.1, 39.8)	21.9	69.3 (54.1, 84.1)	84.9 (40.2, 100.0)	-15.6
Fatty acids	35.9 (29.7, 42.2)	40.6 (34.4, 47.1)	-4.7	51.6 (43.9, 60.3)	44.8 (34.7, 55.7)	6.8	40.3 (35.0, 45.7)	35.4 (19.8, 53.7)	4.9
Moderation components: Higher scores reflect lower concentrations in students' 24 h intakes									
Refined grains	63.0 (56.0, 69.9)	42.4 (33.4, 51.1)	20.6*	65.9 (59.9, 72.0)	47.3 (24.7, 71.2)	18.7	53.7 (45.8, 61.6)	46.9 (35.5, 57.8)	6.9
Sodium	50.8 (46.2, 55.2)	48.0 (40.3, 55.5)	2.8	46.0 (38.9, 53.1)	38.7 (27.6, 50.2)	7.3	42.7 (37.6, 47.8)	44.9 (35.0, 54.8)	-2.2
Empty calories	68.2 (62.9, 73.4)	66.8 (62.7, 71.2)	1.3	70.4 (62.8, 78.5)	62.8 (52.0, 73.2)	7.6	79.9 (76.0, 83.6)	72.9 (64.3, 82.6)	7.0
Total HEI score	64.0 (61.0, 66.8)	59.6 (56.2, 62.8)	4.4*	63.3 (60.6, 65.7)	57.1 (50.4, 63.7)	6.3	67.4 (64.7, 69.9)	62.1 (57.2, 67.3)	5.2

Notes: Mean HEI-2010 scores are expressed as a percentage of maximum possible scores. Upper and lower limits of 95% CIs are included in parentheses below the mean HEI score. * Within a race/ethnicity subgroup, the difference between NSLP participants and nonparticipants was significantly different from zero at the $p < 0.05$ level. CI = confidence interval; HEI = Healthy Eating Index; NSLP = National School Lunch Program. .
